# Thermal and flow dynamics of an inclined air heat exchanger equipped with spring turbulators in the transition flow regime

**DOI:** 10.1038/s41598-024-75337-w

**Published:** 2024-11-12

**Authors:** Devendra Kumar Vishwakarma, Suvanjan Bhattacharyya, Manoj K. Soni

**Affiliations:** 1 Department of Mechanical Engineering, BITS Pilani, Pilani Campus, Pilani, RJ 333031 India; 2 Center of Excellence for Electronics Cooling and Computational Fluid Dynamics Simulation Lab, SRMIST, SRM Nagar, Kattankulathur, Chengalpattu District, TN 603 203 India

**Keywords:** Inclination, Air heat exchanger, Spring turbulators, Transition flow regime, Mechanical engineering, Energy harvesting

## Abstract

The research involves an experimental investigation into the performance of a flow assisting air heat exchanger under varying angular orientation and uniform external heat fluxes without and with spring turbulators. The investigation was performed for Reynolds numbers ranging from 511 to 9676 and inclination angle 15° and 30°. Three heat fluxes (2, 3, and 4 kW/m^2^) were applied to the test section to investigate the effect of external surface heating on the range of transition flow regime and thermohydraulic performance. Transition from laminar to turbulent flow for plain channel at different heat fluxes and inclinations occurs within specific Reynolds number ranges: 2436–4446 for 15° inclination at 4 kW/m^2^, 2574–4289 at 3 kW/m^2^, and 2850–4152 at 2 kW/m^2^; for 30° inclination, the ranges are 2518–4151, 2712–4361, and 2992–4346 at the respective heat fluxes. When it comes to the effect of inclination on Nusselt number, the transition occurs sooner at lower angles, but is delayed as the angle increases. Additionally, the Nusselt number decreases as the angle of inclination increases. When comparing the Nusselt numbers of plain tubes to those with spring turbulators, the latter shows a significantly greater enhancement. In laminar flow, a maximum 100% deviation exists between highest and lowest friction factors, decreasing to 75% with increasing Reynolds number; all insert configurations exhibit highest friction factor at 15° due to stronger buoyancy forces.

## Introduction

Heat exchangers are used to effectively transfer or exchange thermal energy between two or more than two fluids^1^. Their application area is very broad which covers HVAC, power generation, defense, automation, space exploration, pharmaceutical, and so on. Modern heat exchangers have evolved from their predecessors by continuous effort and extensive research by scientists, engineers, and researchers. There are several types of heat exchangers available in the market depending upon the application, namely, shell and tube, tube in tube, spiral, plate type, micro-channel heat exchangers or MCHE, etc^2^. A continuous demand for more efficient, less spacious, and cost-effective heat exchanger keeps the research in the field very active. Features such as heat transfer surface area; mass flow rate; temperature gradient between cold fluid and hot fluid; flow type (laminar or turbulent); fluid properties; insulation; fouling or scaling; design and maintenance; positioning; etc. are the most important in determining the performance of a heat exchanger^3–7^. Hence, analyzing the heat exchangers for their hydro-thermal characteristics is very challenging and requires careful attention.

Heat exchangers are sometime placed in inclined positions to improve the flow stability^8^, efficient cooling^9^ or to accommodate space constraints^10^. By changing the orientation of the heat exchangers, a significant change in its performance takes place, which needs to be properly addressed^11^. Buoyancy force plays a significant role depending upon the orientation of heat exchanger along with mass flow rate^12^. Buoyancy forces are dominant at lower Reynolds number^13^. However, their effect diminishes at higher Reynolds numbers. Inclined or vertical heat exchangers experience mixed (combination of forced and free) convection if the flow rate is very slow. Because of the temperature gradient between the fluid adjacent to the heated surface and the cooler fluid closer to the centerline, buoyancy induced by inclination leads to mixed convection heat transfer^14–18^. Therefore, it is crucial to comprehend the thermophysical properties of the working fluid when heat exchangers are positioned at varying inclination angles.

Buoyancy plays a significant part in the heat transfer process when a heat exchanger is inclined. Specifically, buoyancy impacts the flow of the fluid and the rate of heat transfer between the hot and cold fluids in an inclined heat exchanger^19^. Mixed convection occurs when the heat exchanger is inclined due to the difference in densities of the hot and cold fluids at low Reynolds numbers. However, at higher inclination angles, flow disruption or backflow may take place depending upon the velocity of flow^20^. Sometimes, it also causes thermal stratification in the fluid domain which leads to lower heat transfer effectiveness. It is important to note that inclined heat exchanger design and optimization call for careful consideration of elements such as fluid characteristics, flow rates, heat transfer coefficients, and intended performance goals^21^. Inclining the heat exchangers causes a decrease in the strength of buoyancy forces. Decreased intensity of buoyancy forces resulted in lesser disturbances. As a result of this, both heat transfer and pressure drop decrease^11^. The angle of inclination affects flow patterns and frictional pressure drop. The greatest pressure drop occurs with vertical downward flow, while the lowest occurs with nearly horizontal or vertical upward flow depending on vapor quality^22^.

To this date, heat exchangers are designed to administer in the laminar or turbulent flow regime. No textbook or Databook ever suggested operating a heat exchanger in transition flow. The reason can be unpredictable, chaotic, unsteady, or unrealistic nature of fluid flow in transition flow regime^23^. Although recently some of the researchers claimed that the working range of transition flow regime (2000 ≤ Re ≤ 4000) is irregular^24^ and depends upon various features such as (i) heating or cooling of fluid, (ii) surface geometry (iii) surface roughness (iv) inserts or turbulators, (v) fluid properties, etc^25^. These researchers emphasized the further exploration of above-mentioned parameters for better understanding of flow regimes.

Numerous research works have been carried out to understand the hydro-thermal behavior and overall performance of heat exchangers. A study by Tian et al.^26^ investigated the impact of buoyancy on heat transfer in restricted rectangular channels with uneven heating. It was found that transverse flow and buoyancy forces acting perpendicular to the test-section wall played a significant role in enhancing mixed convection and heat transfer within the channel. Vliet^27^ and Fuji and Imura^28^ made adjustments to account for inclination effects by replacing the gravitational acceleration in the buoyancy force with the buoyancy force component parallel to the vertical surface. Tawfeeq et al.^29^ examined mixed convection heat transfer in inclined tubes of circular cross section under constant wall heat flux boundary condition. The results showed that the Nusselt number increased with higher heat flux and a decrease in tube inclination angle. The maximum Nusselt number occurred at a 30° inclination angle. Tahseen^30^ conducted an experimental study to measure mixed convection in filled circular tubes with a porous media. The experiments showed that the surface temperature of the tube is proportional to its length, and heat transfer occurs through free convection for small Peclet numbers, forced convection for large Peclet numbers, and mixed convection for medium Peclet numbers. Mare et al.^31^ used axial and radial velocity components to measure the mixed convection in inclined isothermal tubes. A study by Kanematsu and Murakami^32^ explored the effects of various inclinations of heat exchangers in a wind tunnel. They tested angles of 0, 45, 60, and 80 degrees and found that the pressure drop experienced a substantial increase between 60 and 80 degrees of inclination.

The past two decades have been dedicated to the profound transition in research, technology, and development all over the world. The size of most of the equipment has been reduced to an impressive scale. However, the advancement in technology generates the need for increased production in industries. Proper space utilization for placement of machines in industries has become necessary to acquire more machines and equipment to satisfy increased production demands. As mentioned above, investigations which include inclination are limited and require immediate attention. Alongside, the impact of transition flow regime on machine performance is understudied and calls for more detailed exploration. Furthermore, the previous studies in the single-phase transition flow regime in the presence of turbulators are very limited and need more attention. Therefore, the main contribution of this experimental investigation is to understand the influence of inclination of air heat exchanger on the thermohydraulic characteristics. The outcomes of this study help the scientific community in better understanding of the working of air heat exchangers in inclined positions with and without spring turbulators.

The outcomes of this study help the scientific community in better understanding the working of air heat exchangers in inclined positions. This includes insights into the thermal performance, pressure drop characteristics, and overall efficiency of the systems under different operational conditions. By doing so, it addresses the critical need for optimizing the design and application of air heat exchangers in various industrial settings, thereby contributing to advancements in energy efficiency and system performance.

Moreover, this research not only enhances the theoretical understanding but also provides practical implications for the design and optimization of air heat exchangers. The findings can be utilized by engineers and designers to develop more efficient and effective heat exchange systems that can operate under various inclined configurations. This could lead to innovations in the deployment of these systems in constrained or unconventional spaces, ultimately broadening the scope of their application and improving their adaptability in real-world scenarios.

Spring tape turbulators are advantageous in heat exchanger use for various reasons. One key benefit is their capacity to increase heat transfer by boosting turbulence in the flow. This leads to better mixing of the fluid, improving heat exchange efficiency between the fluid and heat exchanger surfaces, even during changing flow conditions. Furthermore, spring tape turbulators are versatile, working well in single-phase and two-phase flow situations, making them suitable for various industrial settings. They can also help in designing more compact heat exchangers due to their improved heat transfer, resulting in smaller units that save space and cut material expenses. Additionally, they are cost-effective, being relatively low-priced and simple to install in comparison to other methods of enhancing heat transfer. Along with that, spring tape turbulators have limitations including increased pressure drop, potential fouling, mechanical stability concerns, and added manufacturing complexity. These drawbacks can offset efficiency gains and may not be beneficial for certain applications or fluids, such as highly viscous fluids. However, previous studies utilizing similar applications and turbulators have yielded promising results, which prompted the initiation of the present investigation^33–35^.

## Experimental methodology

**Fig. 1:**
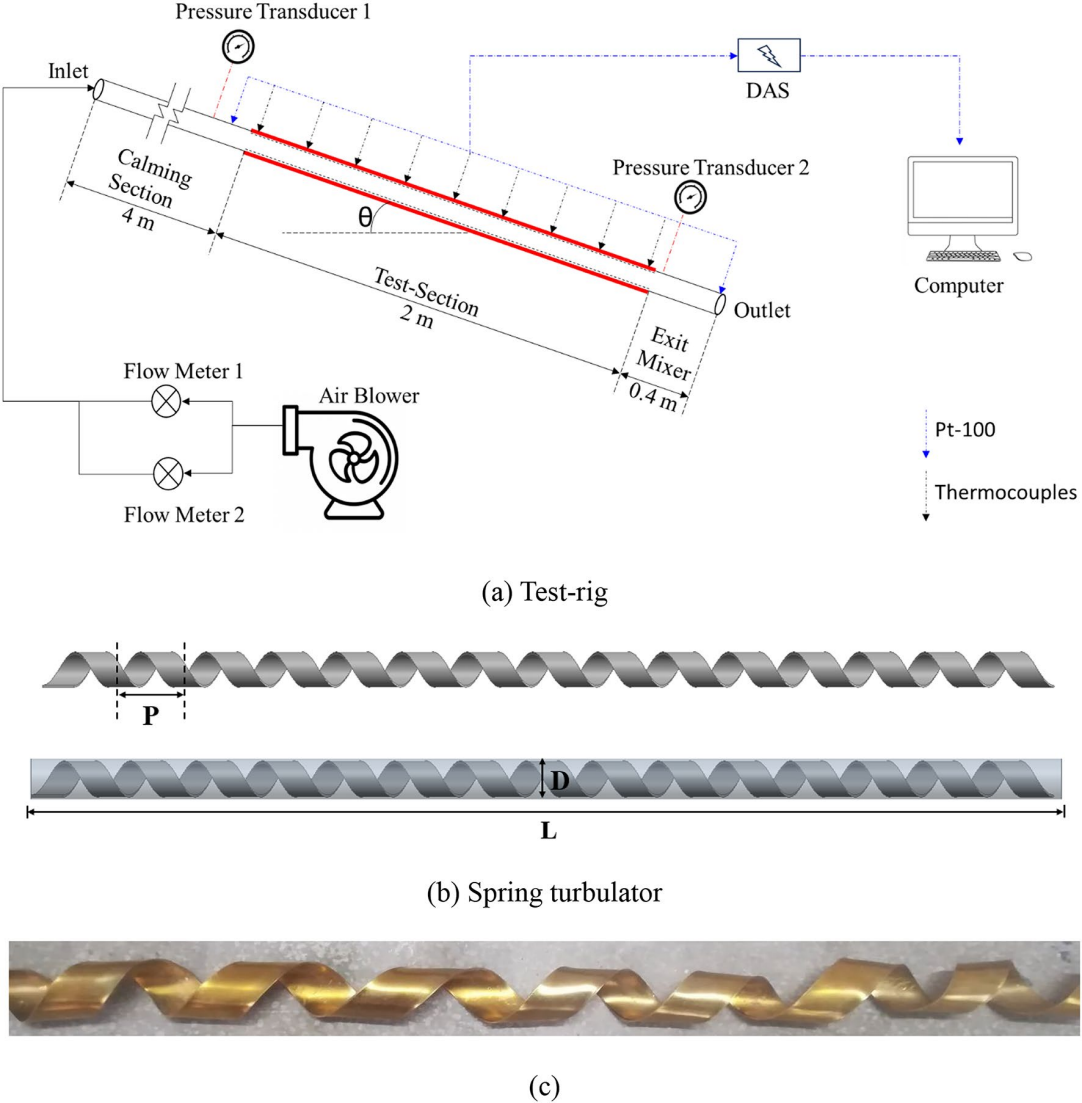
Schematic of (**a**) Test-rig, and (**b**) Spring turbulator.

Figure 1 shows the schematic of experimental unit and spring turbulator used in the investigation. It consists of a 7-kW power capacity air blower. Air blower is connected with two rotameters. The flow through the rotameters is controlled using control valves which are placed right before the rotameters. Rotameters are connected with the calming section using a flexible tube having 20 mm diameter. The flexible tube assists in angular movement of test-section. The calming section is made up of GI pipe of same dimensions as flexible tube. The calming section allows the flow uniformity before it enters the test-section. The test section is made of brass and is 2000 mm long. At the far end of the test section, an outlet mixer is attached which mixes the air leaving the experimental setup.

The test section is facilitated with 36-T type thermocouples at 9 stations and two PT-100 to measure the bulk temperature at inlet and outlet. A u-tube rotameter is also attached to the test section to measure the pressure drop across the test section.

Experiments are carried out in three phases. During phase-I, experiments were carried out with plain horizontal channel (heat flux of 2 kW/m^2^) and results obtained are used for validation of the study. In phase-II, the test section is placed in inclined position and results are used to determine the range of transition flow along with Nusselt number and friction factor for different Reynolds numbers. In the final phase, the test section is inserted with spring turbulators having different spring ratio $\left(S.R.=\frac{Pitch}{Internal\; Diameter}=\frac{P}{D}=3\; or\; 4\; or\; 5\right)$. For all the phases, experiments were conducted for 2, 3 and 4 kW/m^2^ heat fluxes.

The spring tapes used in the process are fabricated in the mechanical workshop. First, the selected material is cut into thin strips of the desired width, which depends on the specific design requirements of the heat exchanger and the dimensions of the tubes. A coiling machine is then used to form the tape into a helical spring shape. The pitch and diameter of the spring are carefully controlled to ensure consistency and effectiveness. The same procedure was repeated for different tapes.

The time it took for the system to stabilize varied depending on the rate at which mass flowed through it, typically taking around 2 to 2.5 h. We determined that the system had reached a steady state when we observed that temperature, pressure (ΔP), and mass flow rate remained within a deviation of 0.1 °C, 1 mm of Hg, and 1 LPM, respectively, for a continuous 10-min period. Once the system was stable, we collected 60 data points within a span of 1 min and calculated the average as a single data value. For further information, interested readers are directed to previously published articles^35–39^.

### Data reduction and uncertainity

Step 1: Determination of bulk temperature of fluid.

1 $${T}_{bulk}=\frac{{T}_{out}+{T}_{in}}{2}$$

Step 2: Determination of local temperature axially.

2 $${T}_{local}=\frac{{T}_{out}-{T}_{in}}{L}x+{T}_{in}$$

Step 3: Determination of ΔP across channel.

3 $$\varDelta\, P=\varDelta\,{P}_{in}-\varDelta\,{P}_{out}$$

Step 4: Determination of *f*_D_.

4 $$f=\frac{2*D*\varDelta\, P}{L*\rho\, *{v}^{2}}$$

Step 5: Determination of Re^40^.

5 $$Re=\frac{4*\dot{m}}{\pi *\mu *D}$$

Step 6: Determination of H.Tran. to the fluid^41^.

6 $$\varnothing = \dot{m}{c}_{p}\left({T}_{out}-{T}_{in}\right)$$

Step 6: Energy balance

7 $$Energy\;Balance\;Error= \frac{{\varnothing }_{e}-\varnothing }{{\varnothing }_{e}}\times 100$$

Step 7: Determination of Heat Flux^42^.

8 $$\dot{\varnothing }=\varnothing /{A}_{s}= \dot{m}{C}_{p}\left({T}_{Out}-{T}_{in}\right)/\pi\, DL$$

Step 8: Determination of H.Tran. coefficient^42^.

9 $$h= \varnothing /({T}_{surface}-{T}_{local})$$

Step 9: Determination of thermal resistance of brass tube^42^.

10 $${R}_{tube}=\frac{lnDo/D}{2\pi {\kappa }_{tube}L}$$

Step 10: Determination of Nusselt number^43^.

11 $$Nu= hD/\kappa$$

Step 11: Determination of thermal performance factor.

12 $$\eta =\frac{\raisebox{1ex}{$Nu$}\!\left/\:\!\raisebox{-1ex}{${Nu}_{o}$}\right.}{{\left(\raisebox{1ex}{$f$}\!\left/\:\!\raisebox{-1ex}{${f}_{o}$}\right.\right)}^{0.33}}$$

The rated accuracy of various measuring instruments is provided in Table 1.

**Table 1:** Rated accuracy of measuring instruments.

S.*N*.	Parameter	Error (%)
1	Thermocouples	0.75
2	Air flow meters	1
3	Variac	0.75
4	Voltmeter	1
5	Ammeter	1
6	Manometer	1

Kline and McClintock^44^ have utilized the method described to ascertain the level of uncertainty. The details regarding the uncertainty related to the different parameters analyzed in this study are presented in Table 2.

**Table 2:** Calculated uncertainty of Re, Nu and *f*.

S.*N*.	Parameter	Uncertainty (δ) (%)
1	Re	1.24
2	*Nu*	1.09
3	*f*	2.04

### Validation

**Fig. 2:**
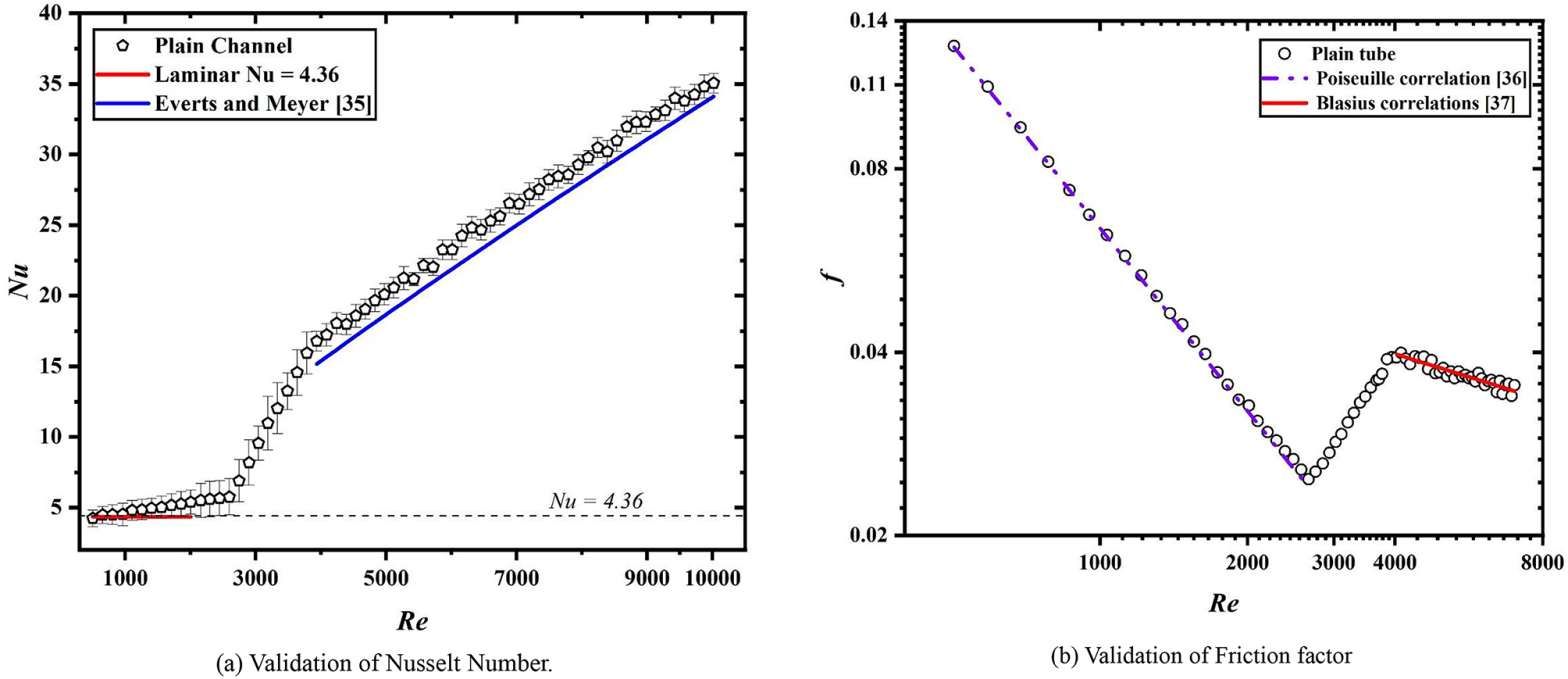
Validation of experiments.

For validating the results, the test-section was kept at 0° inclination from the ground surface. The test-section was kept clear without any modification and the results for Nusselt number and friction factor were noted. The Nusselt number results were validated with Nu = 4.36 in laminar regime and with Meyer et al.^45^ for turbulent flow regime, as shown in Fig. 2a. For friction factor validation (Fig. 2b), the correlation of Poiseuille’s^46^ and Blasius^47^ was used.

## Results and discussion

### Determination of transition flow regime

**Fig. 3:**
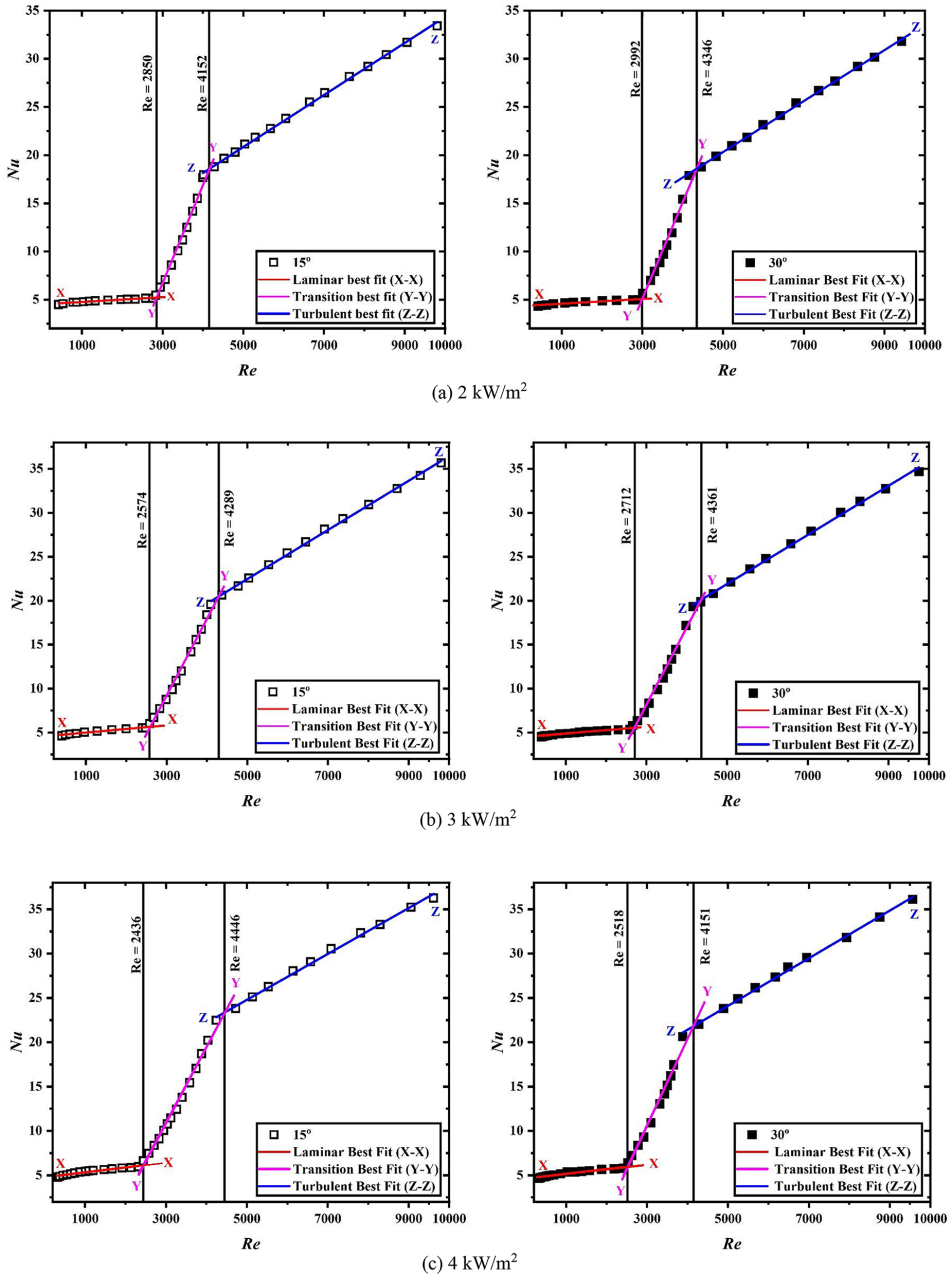
Nusselt number for plain inclined channel at different heat fluxes.

Figure 3a–c provide the Nusselt number as a function of Reynolds number for uniform heat flux condition of 2 kW/m^2^, 3 kW/m^2^ and 4 kW/m^2^, respectively, for plain heated solar air heater tube using linear curve fitting method. Solid square marks used in the figure represent the data for 30° inclination while the blank square marks represent the data associated with 15° inclination. Line X-X represents the laminar best-fit, Y-Y represents the transition best-fit, and Z-Z represents the turbulent best-fit.

For 4 kW/m^2^ applied heat flux at 15° inclination, the transition initiates at Reynolds number 2436 and terminates at Reynolds number 4446. For 3 kW/m^2^, the critical Reynolds number ranges between 2574 and 4289. And for 2 kW/m^2^, the transition initiates at Reynolds number 2850 and terminates at Reynolds number 4152. For 30° inclination, the transition institutes at Reynolds number 2518, 2712, and 2992, and terminates at Reynolds number 4151, 4361, and 4346 for 4kW/m^2^, 3 kW/m^2^ and 2 kW/m^2^, respectively. At higher heat fluxes, the disturbance in the air increases which results in the early start of transition at higher heat fluxes in comparison to lower heat fluxes. As far as the inclination and its influence on Nusselt number is concerned, transition begins early for lower angular position while it delayed with increase in the angle. Also, Nusselt number decreases with increase in the angle of inclination. While the deviation in the Nusselt number may not be substantial, it is distinctly discernible. The deviation of Nusselt number between two angular positions is approx. 3.6%.

**Fig. 4:**
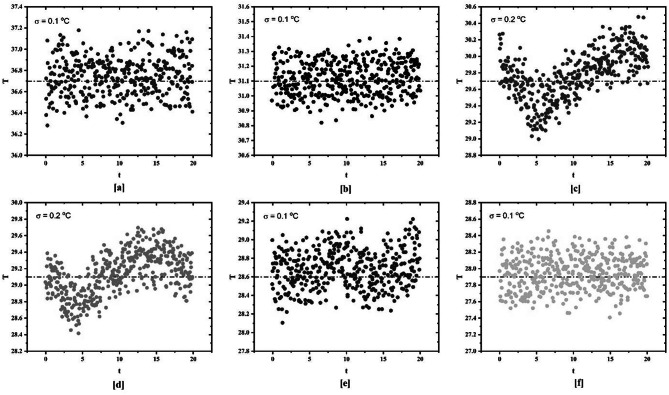
Standard deviation of surface temperature for plain tube at different Reynolds numbers.

Figure 4 provides the standard deviation of temperature data for various Reynolds numbers for plain channel inclined at 15°. The temperature data is taken from station 8 on the test section. The standard deviation is calculated for different Reynolds numbers (6641, 3498, 1331, 871, 526, 413). It has been found that the standard deviation for laminar and turbulent flow regime is 0.1 while the standard deviation for transition flow regime is 0.2. A similar approach is used for determination of transition flow regime when spring turbulators are used which is discussed in the upcoming sections.

### Heat transfer

**Fig. 5:**
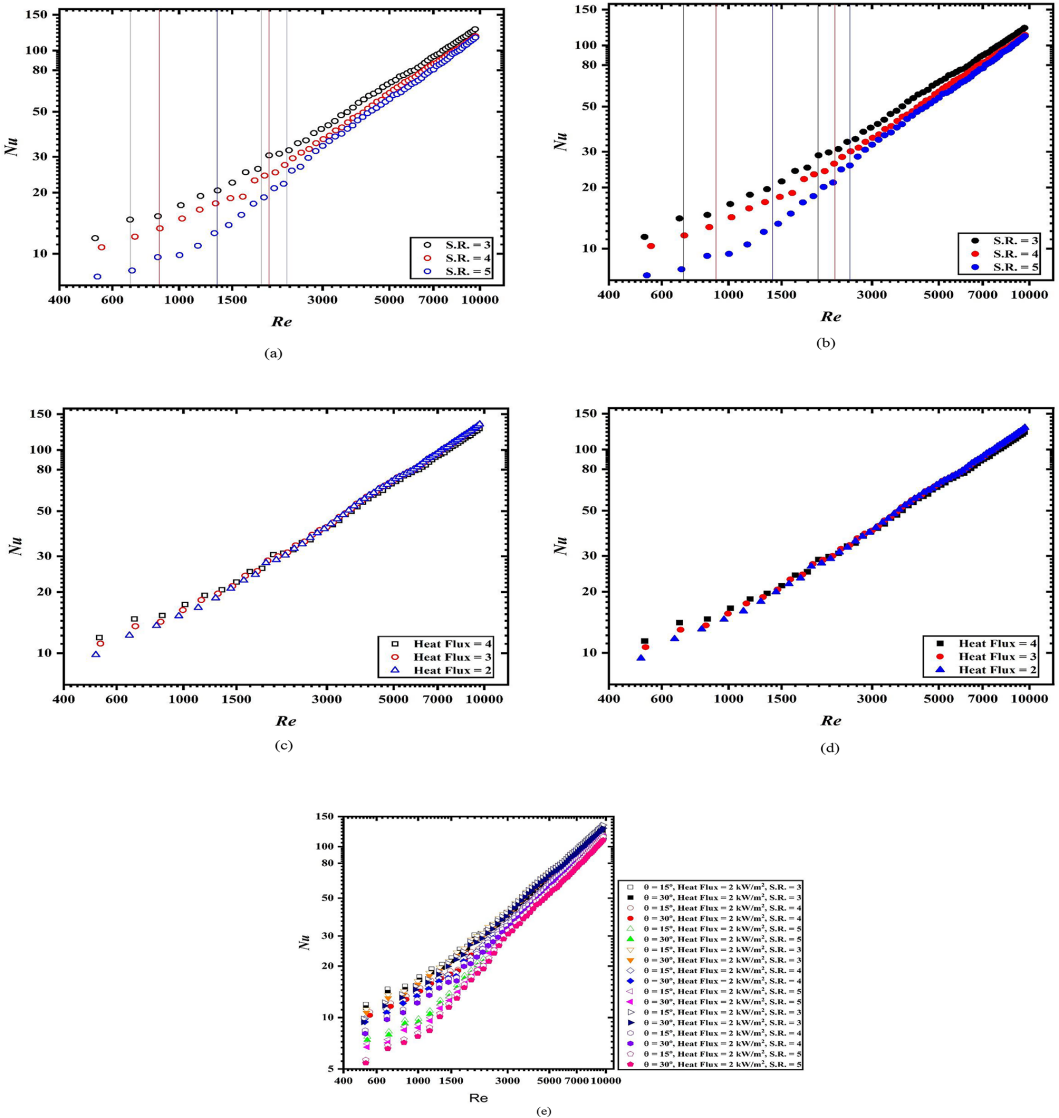
Nusselt number variation with respect of Reynolds number (**a**) 15°, (**b**) 30°, (**c**) 15°, S.R. = 3, (**d**) 30°, S.R. = 3, (**e**) all configuration and operating conditions.

Figure 5a, b provide the Nusselt number for spring turbulators at 15° and 30° inclination, respectively, at a uniform heat flux of 4 kW/m^2^. The black, red and blue color lines indicate the boundaries or width of transition flow regime. As per Fig. 5a, b, the transition begins and terminates earliest for spring turbulators having spring ratio (S.R.) of 3. While the beginning and end of transition delayed most for spring turbulators having spring ratio (S.R.) of 5. At lower spring ratio, the disturbances are higher which resulted in the early embark of the transition while at higher spring ratio the disturbances are less due to larger gap between tapes which resulted in delayed transition.

The transition for 15° inclination started earlier when compared with 30° inclination. This is due to the buoyancy forces. At 15° angular position, the buoyancy forces are higher when compared with the 30°. This resulted in the early start of transition at lower angular orientation.

On comparison with the plain tubes Nusselt numbers, the enhancement in the Nusselt numbers for tube with spring turbulators is significantly larger. The spring turbulators inside the plain tube cause the intense disruption of boundary layers which resulted in the mixing of the fluid. As a result, the enhancement in the Nusselt number is obtained. The Nusselt numbers for 15° inclination are approximately 4% larger than the Nusselt numbers of 30° inclination for different Reynolds numbers.

Figure 5c, d depict the Nusselt numbers as a function of Reynolds number for uniform spring ratio (S.R.) of 3 while varying the applied heat fluxes for 15° and 30° inclination, respectively. It is found that the Nusselt numbers are highest for case when 4 kW/m^2^ of heat flux is applied. Also, for 15°, the Nusselt number is slightly higher than the Nusselt numbers for 30° inclination. The variation of Nusselt numbers in the laminar and turbulent flow regime is clear while in the turbulent flow regime, it is not discernible. The results obtained from the present investigation are in line with the results obtained from the Vishwakarma et al.^35^ for horizontal channel.

Figure 5e showed the Nusselt number variation for all the cases for all the operating conditions. From the figure, it is clear that the Nusselt number remains highest for S.R. equals to 3 while remain lowest for S.R. equals to 5 at uniform heat fluxes. Also, at higher heat flux, the Nusselt number remains higher when compared with lower heat fluxes. On comparing the lowest Nusselt number (at θ = 30°, Heat flux = 2 kW/m^2^, S.R. = 5), 100% increment is noticed (at θ = 15°, Heat flux = 4 kW/m^2^, S.R. = 3). The percentage enhancement in the Nusselt number is significantly higher in the turbulent region. However, the percentage increase in lower in this regime.

**Fig. 6:**
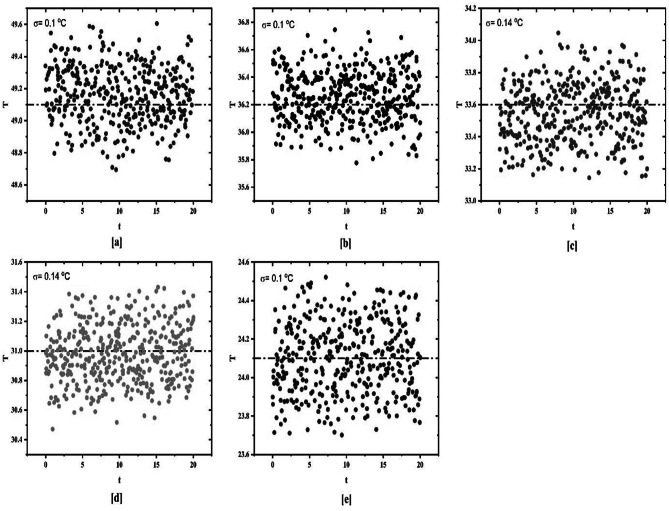
Standard deviation of surface temperature for plain tube with spring turbulators at different Reynolds numbers for 4 kW/m^2^ heat flux.

For determination of transition flow regime, linear curve fitting method showed insignificant results for tube inserted with spring turbulators. Hence, standard deviation method is employed for identification of transition flow regime. On the basis of results obtained from the standard deviation method transition flow regime is determined. Figure 6 reveals the standard deviation of temperature data for plain tube fitted with spring turbulator having spring ratio (S.R.) of 3 while being heated at 4 kW/m^2^. The standard deviation for laminar and turbulent flow regime remains uniform at 0.1 while in transition flow regime, the standard deviation changes to 0.14.

In percentage terms, the Nusselt number for a spring ratio (S.R.) of 3 with an applied heat flux of 4 is 17.75% higher compared to that with a heat flux of 2, given the same spring ratio at 15° inclination. For a uniform heat flux of 4 and varying spring ratios (S.R.), the percentage improvement in the Nusselt number is 35.34%, with higher values observed for lower S.R. The deviation in the Nusselt number is most pronounced in the laminar and transitional flow regimes and diminishes as it approaches the turbulent flow regime.

### Pressure drop

**Fig. 7:**
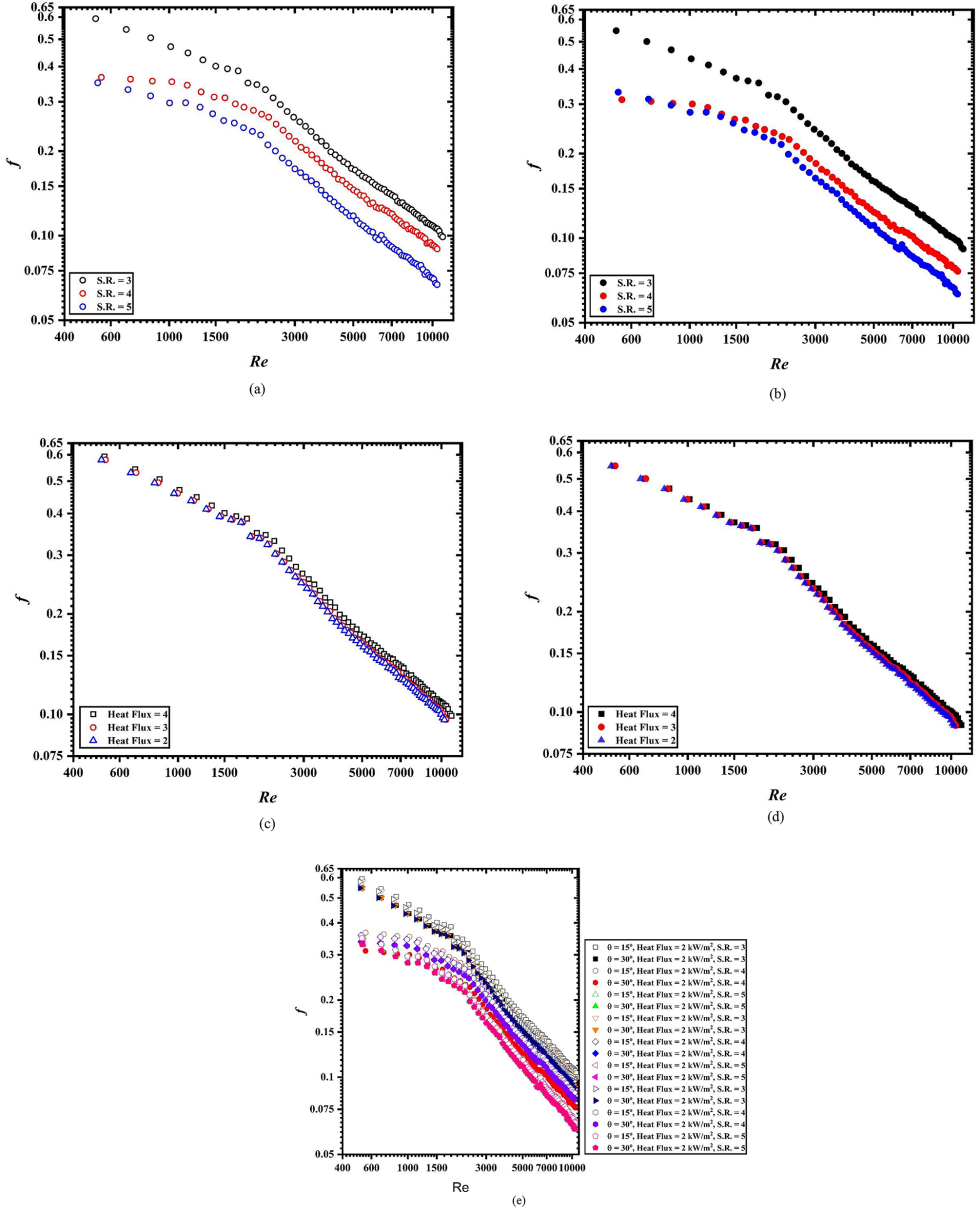
Friction factor variation with respect of Reynolds number (**a**) 15°, (**b**) 30°, (**c**) 15°, S.R. = 3, (**d**) 30°, S.R. = 3, (**e**) all configuration and operating conditions.

Figure 7a–e provide the friction factor in terms of Reynolds numbers for different operating conditions at 15° and 30°, inclination. It is noted from the figures that the friction factor shows a decreasing trend with an increase in the Reynolds number for all the operating conditions. Figure 7a, b shows the friction factor for uniform heat flux (4kW/m^2^) at different S.R. (3–5), for 15° and 30°, respectively. Highest friction is found when spring turbulator having S.R. of 3 while heated with 4 kW/m^2^ of heat flux and inclination of 15° is used (refer Fig. 7a, e). For uniform heat flux conditions, the lowest friction factor is reported when S.R. is 5 (Fig. 7b). This is due to the lower contact surface area for the spring turbulator having S.R. equal to 5. However, with uniform S.R. and varying heat fluxes, the difference in the insignificant and hence is not discernible (Fig. 7c, d).

Figure 7e provides a better clarity of the friction factor and its variation with respect to Reynolds number. A significant change in the friction factor can be observed. A maximum of 100% deviation has been observed between the highest and lowest friction factor in the laminar flow regime. Although, with an increase in the Reynolds number, this percentage deviation starts to decrease (75%). For all the configurations of inserts, the highest friction factor was reported with 15°. This is due to the higher buoyancy forces at lower inclination.

### Thermal performance factor

**Fig. 8:**
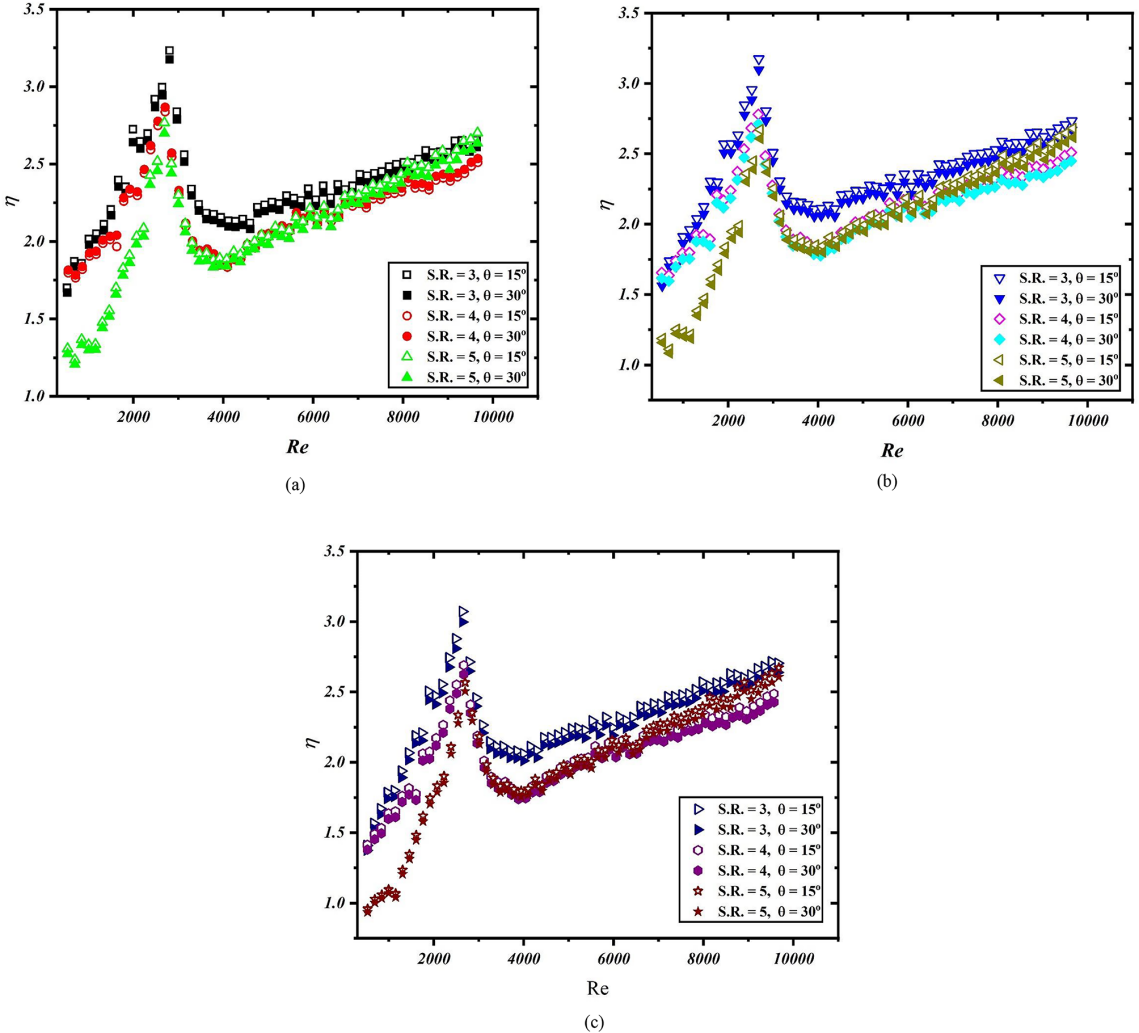
Thermal performance factor with respect to Reynolds number (**a**) at q = 4, (**b**) at q = 3, and (**c**) at q = 2.

To facilitate a clearer understanding of the results of heat transfer enhancement, a non-dimensional parameter known as the thermal performance factor, denoted by ‘η’, is utilized. This factor is defined as the ratio of the Nusselt number to the friction factor, offering an indication of the heat transfer enhancement. A value of η greater than unity suggests that the enhancement in heat transfer surpasses the increase in the friction factor. Figure 8a–c indicate the variation of thermal performance factor against the Reynolds number for applied heat flux of 4, 3 and 2 kW/m^2^, respectively. The hollow markers indicate the 15° inclination while the solid markers indicate the 30° inclination. Thermal performance remains highest when heat flux is higher while reduce to lowest with decrease in the heat flux. For 15° inclination, the thermal performance remains higher on comparison to 30° inclination for individual spring ratio. It has been found that thermal performance remains lowest in the laminar flow regime and increases with Reynolds number up to approx. Re = 3000. Beyond this point the value of thermal performance again decreases up to approx. Re = 4000. A further momentum gain in the thermal performance was noticed beyond Re = 4000. For all the cases involved, the value of thermal performance factor remains higher than unity which indicates that the present configuration of spring tape turbulator and solar air heater are feasible and can be further used. From the above discussion, it can be said that the present configuration is best if operated in transition and turbulent flow regime.

### Correlations

**Fig. 9:**
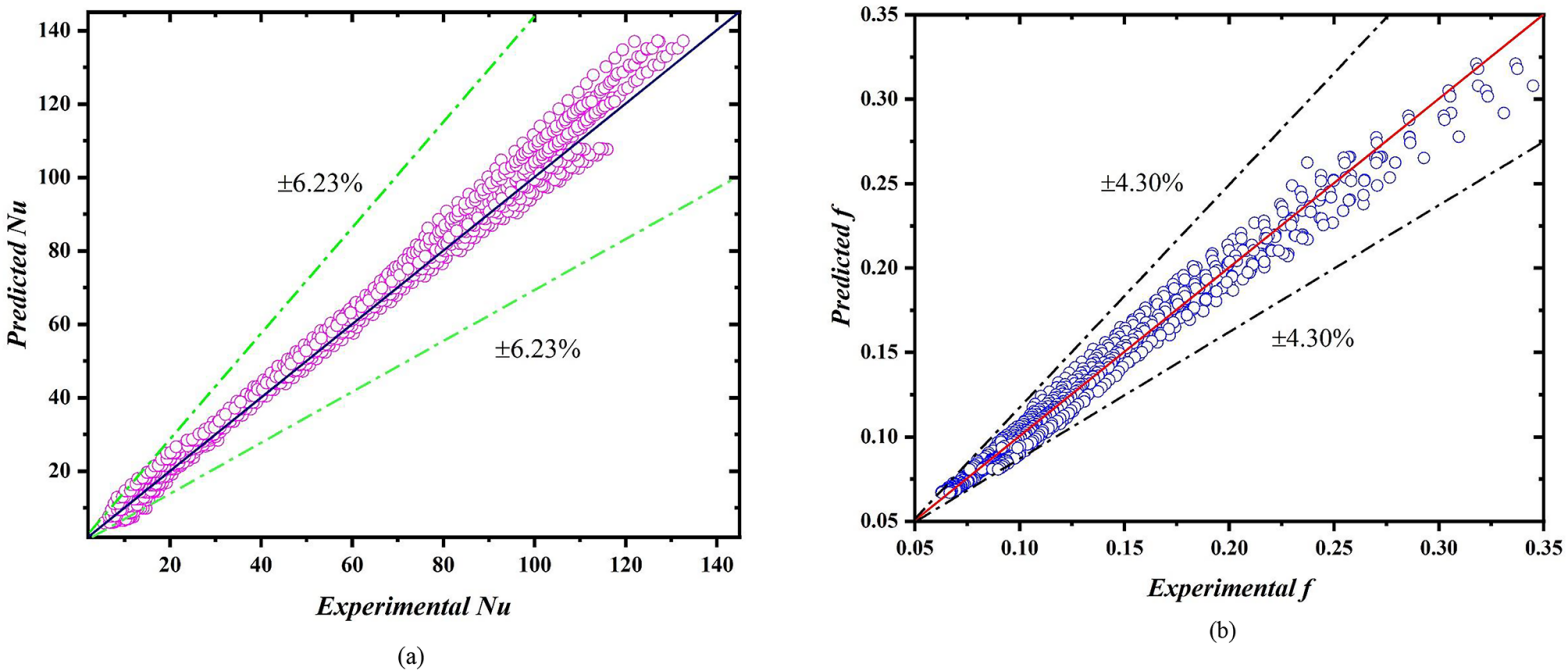
Deviation between experimental data and predicted data (**a**) Nu, (**b**) *f*.

The following correlations are developed using the experimentally obtained data to predict the Nusselt number (Eq. 13) and friction factor (Eq. 14) for unknown range of parameters. Novel correlations generated using regression analysis in Microsoft Excel software. The developed correlations are in very good agreement with the experimental data. The average deviation of Nusselt number is ± 6.23% while the average deviation of friction factor is ± 4.30%. Figure 9a, b represent the average deviation between experimental and predicted data for Nusselt number and friction factor respectively.

13 $$\begin{aligned} Nu &= \left[\right\{(-0.006\times\, \text{ln}\left(S.R.\right)+0.0208)\times\, Re\}+0.0436] \\ &\quad 400 \le Re \le 20{,}000 \end{aligned}$$

14 $$\begin{aligned} f &=[149.12\times\, \text{e}\text{x}\text{p}(-0.183\times\, S.R.)\times {Re}^{-0.734}]\\ &\quad 400 \le Re \le 20{,}000 \end{aligned}$$

### Comparison analysis

**Fig. 10:**
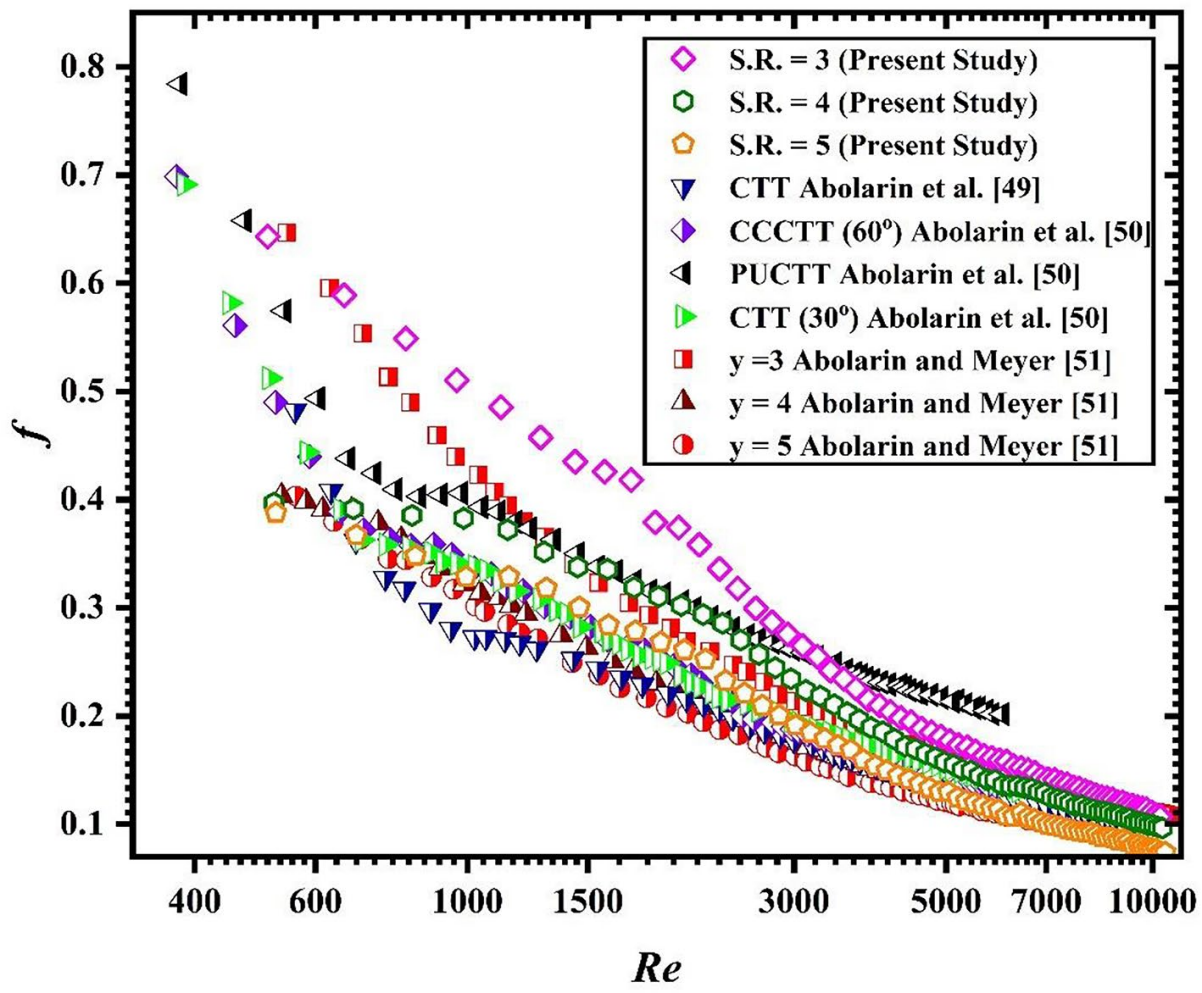
Comparison of friction factor of present study with published literature.

The experimental results of friction factor obtained in the present study are compared with those of previously published articles in different flow regimes. The results of present investigations are compared with that of Abolarin et al.^48,49^ and Meyer and Abolarin^50^. Figure 10 shows the graphical representation of comparison of friction factor of present investigation with previous studies.

## Conclusion

The transition begins and terminates earliest for spring turbulator having spring ratio (S.R.) of 3. While the beginning and end of transition delayed most for spring turbulator having spring ratio (S.R.) of 5. The transition for 15° inclination started earlier when compared with 30° inclination. On Comparison with the plain tubes Nusselt numbers, the enhancement in the Nusselt numbers for tube with spring turbulators is significantly larger. The Nusselt numbers for 15° inclination are approximately 4% larger than the Nusselt numbers of 30° inclination for different Reynolds numbers. On comparing the lowest Nusselt number (at θ = 30°, Heat flux = 2 kW/m^2^, S.R. = 5), 100% increment is noticed (at θ = 15°, Heat flux = 4 kW/m^2^, S.R. = 3).

## Data Availability

The datasets used and/or analysed during the current study available from the corresponding author on reasonable request.
